# Draft Genome Sequence of *Chromobacterium subtsugae* MWU12-2387 Isolated from a Wild Cranberry Bog in Truro, Massachusetts

**DOI:** 10.1128/genomeA.01633-16

**Published:** 2017-03-23

**Authors:** Kristin Vöing, Alisha Harrison, Scott D. Soby

**Affiliations:** a Fachbereich Biologie, Universität Münster, Münster, Germany; b Biomedical Sciences Program, College of Health Sciences and College of Veterinary Medicine, Midwestern University, Glendale, Arizona, USA

## Abstract

*Chromobacterium subtsugae* MWU12-2387 was isolated from the rhizosphere of cranberry plants. While it is unknown what environmental role these bacteria play in bog soils, they hold potential as biological control agents against nematodes and insect pests. Potential virulence genes were identified, including the violacein synthesis pathway, siderophores, and several chitinases.

## GENOME ANNOUNCEMENT

*Chromobacterium subtsugae* strain MWU12-2387 was isolated from the roots of wild cranberry plants in Truro, MA, and tentatively identified as *C. subtsugae* by phenotype and by 16S rRNA sequence (1–3). *C. subtsugae* has insecticidal properties, most likely due to multiple virulence mechanisms (4). Its genome was sequenced at the Arizona State University CLAS Genomics Core facility using an Illumina MiSeq. Genomic DNA was sheared to approximately 600-bp fragments using the Covaris M220 ultrasonicator, and Illumina libraries were generated on an Apollo 384 liquid handler (Wafergen) using a Kapa Biosystems library preparation kit (catalog no. KK8201). DNA fragments were end-repaired and A-tailed as described in the Kapa protocol. Combined indexes/adapters (catalog no. 520999; Bioo) were ligated onto each sample and multiplexed into one lane. Adapter-ligated molecules were cleaned using AMPure beads (catalog no. A63883; Agencourt Bioscience/Beckman Coulter, Inc.) and amplified with Kapa HIFI enzyme. Libraries were analyzed on an Agilent Bioanalyzer and quantified by quantitative PCR (qPCR) (catalog no. KK4835; Kapa library quantification kit) before multiplex pooling and sequencing in a 2 × 300 paired-end (PE) flow cell on the MiSeq platform (Illumina). Adapters were computationally segregated and trimmed in the Illumina BaseSpace pipeline. The Velvet assembly tool (BaseSpace) was used for signal processing and partial sequence assembly. The sequence is 64.8% G + C and consists of 4,788,922 bp distributed over 243 scaffolds, 129 of which are larger than 1 kbp. The largest contig is 184,525 bp, the *N*_50_ is 89,418 bp, and the *N*_75_ is 42,761 bp, with a sequence coverage of 45.75×. The isolate MWU12-2387 genome sequence was compared to reference genomes of *Chromobacterium violaceum* (ATCC 12472), *Chromobacterium haemolyticum* (T124), *Chromobacterium vaccinii* (MWU205), *Chromobacterium pseudoviolaceum* (LMG 3953), *Chromobacterium aquaticum* (CC-SEYA-1), and *C. subtsugae* (F49) using the Genome-to-Genome Distance Calculator (GGDC) provided online by the DSMZ. GGDC mimics *in vitro* DNA-DNA hybridization by dividing scaffold sequences into fragments approximately the same size as would be expected *in vitro*, and by pairing up homologous segments (5–7). The MWU12-2387 genome was 90% homologous to a *C. subtsugae* reference genome, confirming it as a member of this species.

*Ab initio* gene prediction was performed on the assembly using RAST (http://rast.nmpdr.org/). A number of potential virulence factor genes were found that may contribute to insect toxicity, including production of the pigment violacein (8), homologs of *Mycobacterium* virulence operons (9), nonribosomal peptide synthesis siderophores, hydrogen cyanide (10), type III secretion system-associated effectors, chitin binding protein, and secreted chitinases (11, 12). MWU2387 contains 15 probable chitinase genes, including four probable chitinase A genes and 10 endochitinases.

### Accession number(s).

This whole-genome shotgun project has been deposited at DDBJ/EMBL/GenBank under the accession number MQZZ00000000. The version described in this paper is version MQZZ01000000.
